# Characterization and Genome Analysis of Vibrio campbellii Lytic Bacteriophage OPA17

**DOI:** 10.1128/spectrum.01623-22

**Published:** 2023-01-31

**Authors:** Intraporn Srisangthong, Chadtida Sangseedum, Netnapa Chaichanit, Komwit Surachat, Naraid Suanyuk, Pimonsri Mittraparp-arthorn

**Affiliations:** a Division of Biological Science, Faculty of Science, Prince of Songkla University, Hat Yai, Songkhla, Thailand; b Molecular Evolution and Computational Biology Research Unit, Faculty of Science, Prince of Songkla University, Hat Yai, Songkhla, Thailand; c Division of Computational Science, Faculty of Science, Prince of Songkla University, Hat Yai, Songkhla, Thailand; d Aquatic Science and Innovative Management Division, Faculty of Natural Resources, Prince of Songkla University, Hat Yai, Songkhla, Thailand; Institut Pasteur

**Keywords:** Aquaculture, bacteriophage, biocontrol, biofilm, luminous vibriosis, *Siphoviridae*, *V. campbellii*

## Abstract

Vibrio campbellii is a marine bacterium that is associated with luminous vibriosis, especially in the hatchery and nursery stages of penaeid shrimp cultivation worldwide, which has led to low survival rates of shrimp during aquaculture. Phage therapy has been reported as an alternative biocontrol agent which can reduce or replace the use of antibiotics and other chemicals. This study characterized a lytic *V. campbellii* bacteriophage, OPA17, originally isolated from bloody clams and investigated its biocontrol efficacy against *V. campbellii* infection in a model system, Artemia franciscana. Phage OPA17 lysed 83.89% of *V. campbellii* strains tested (*n *= 118) with clear plaque morphology. Some strains of Vibrio parahaemolyticus and Vibrio vulnificus were also infected by phage OPA17. Transmission electron microscopy and genetic features indicated that OPA17 belongs to the *Siphoviridae* family. The latent period and burst size of OPA17 were approximately 50 min and 123 PFU/cell, respectively. Moreover, it survived in artificial seawater throughout the 2-month study period and effectively destroyed Vibrio campbellii biofilms after 4 h of incubation. The addition of OPA17 significantly increased the survival of *A. franciscana* nauplii infected with *V. campbellii*. The genome sequence of OPA17 showed that it does not carry genes unsuitable for phage therapy. The phylogenetic tree analysis showed that OPA17 was closely related to the V. vulnificus lytic phage SSP002 (98.90% similarity), which was previously reported as a potential biocontrol agent. Accordingly, the results of this study provide valuable information regarding the potential biocontrol application of phage OPA17 against *V. campbellii*.

**IMPORTANCE** *V. campbellii* is an emerging luminous pathogen associated with vibriosis, especially in marine shrimp hatcheries. Several strategies, including pond management and use of natural antimicrobials and probiotics, have been studied for control of this organism. Phage therapy is considered one of the effective biocontrol strategies against bacterial infections in aquaculture. However, there has been limited study of *V. campbellii* bacteriophages. In this study, *V. campbellii*-specific bacteriophage OPA17 was isolated, characterized, and investigated for its biocontrol efficacy against *V. campbellii* infection in an *Artemia* nauplii model. Phage OPA17 belongs to the *Siphoviridae* family and shares significant genome similarity to phage SSP002, a potential biocontrol agent against V. vulnificus infection in a murine model. However, the host range of OPA17 was broader than that of SSP002. Overall, we discuss the potential of OPA17 for phage therapy application in shrimp hatcheries.

## INTRODUCTION

Bacteria in the genus *Vibrio* can be found in marine, estuarine, and freshwater environments. Many species are well known to cause diseases in fish, shellfish, and mammals, as well as in humans (1, 2). Vibrio campbellii is considered an emerging opportunistic pathogen which is responsible for luminous vibriosis, especially in marine shrimp hatcheries (3–5). It belongs in the Harveyi clade, which is composed of various *Vibrio* species, including V. harveyi, V. campbellii, V. alginolyticus, V. rotiferianus, V. parahaemolyticus, V. natriegens, V. mytili, and V. azureus (6). The pathogenicity and virulence of these species have been reported (7).

*V. campbellii* is closely related to V. harveyi, and they share more than 97% similarity in their 16S rRNA gene (8). Thus, the diseases caused by *V. campbellii* were underreported because of misidentification between these two species. Outbreaks of luminous vibriosis have been reported in penaeid shrimp worldwide, which led to low survival rates of shrimp in hatcheries and grow-out ponds (5, 9–11). Antibiotics have been used to treat and control bacterial infection in shrimp aquaculture; however, extensive use of antibiotics leads to their residues and resistant bacterial strains (12), which becomes a global problem. In addition, *Vibrio* spp. can form biofilms, causing problems with the antibiotic treatment (13). Thus, various biocontrol strategies have been studied for sustainable aquaculture, and phage-based biocontrol has been suggested as a potential method to treat the antibiotic-resistant pathogen and biofilms in the postantibiotic era (14–16).

Bacteriophages are viruses that specifically infect and destroy target bacteria. There has been an increasing amount of research focused on the use of bacteriophages in aquaculture to prevent infection or kill pathogenic bacteria. For examples, the lytic bacteriophages designated uVh1, uVh2, uVh3, and uVh4 were isolated from commercial shrimp hatcheries and are considered potential biocontrol agents for V. harveyi in shrimp hatcheries (17). The ability of phage VHP6b to protect postlarval shrimp against V. harveyi infection was demonstrated (18). Moreover, phages A3S and Vpms1 were shown to reduce mortality in shrimp larvae caused by V. parahaemolyticus (19). Phage vB_VpaP_MGD2 was suggested as a biocontrol agent against acute hepatopancreatic necrosis disease (AHPND) caused by multidrug-resistant V. parahaemolyticus (20, 21). V. vulnificus phages VV1, VV2, VV3, and VV4 have a broad lytic spectrum and potential for biocontrol of vibriosis in the shrimp aquaculture (22). Phage Φ-5 was shown to be a potential biocontrol agent against V. alginolyticus infection in oyster (Saccostrea glomerata) larvae (23). V. alginolyticus phages φSt2 and φGrn were shown to reduce the *Vibrio* load in Artemia salina, zooplankton used as live feed in hatcheries (24). In *V. campbellii*, phages vB_Vc_SrVc2 and vB_Vc_SrVc9 were demonstrated against *V. campbellii* infection in *A. franciscana* larvae (4, 25). In addition, vB_VcaS_HC has been isolated and characterized as a potential candidate for *V. campbellii* control. Our previous study reported the isolation and host range of OPB45, OPB48, OPB54, OPB58, and OPB66 lytic phages against *V. campbellii* (26). In this study, we therefore isolated a larger number of *V. campbellii* hosts, searched for the effective phage that could inhibit planktonic cells and destroy bacterial biofilm, and performed genome sequencing, thus, making these phages potentially excellent candidates for biocontrol against *V. campbellii* infection.

## RESULTS

### Isolation and morphological characteristics of phage OPA17.

**FIG 1:**
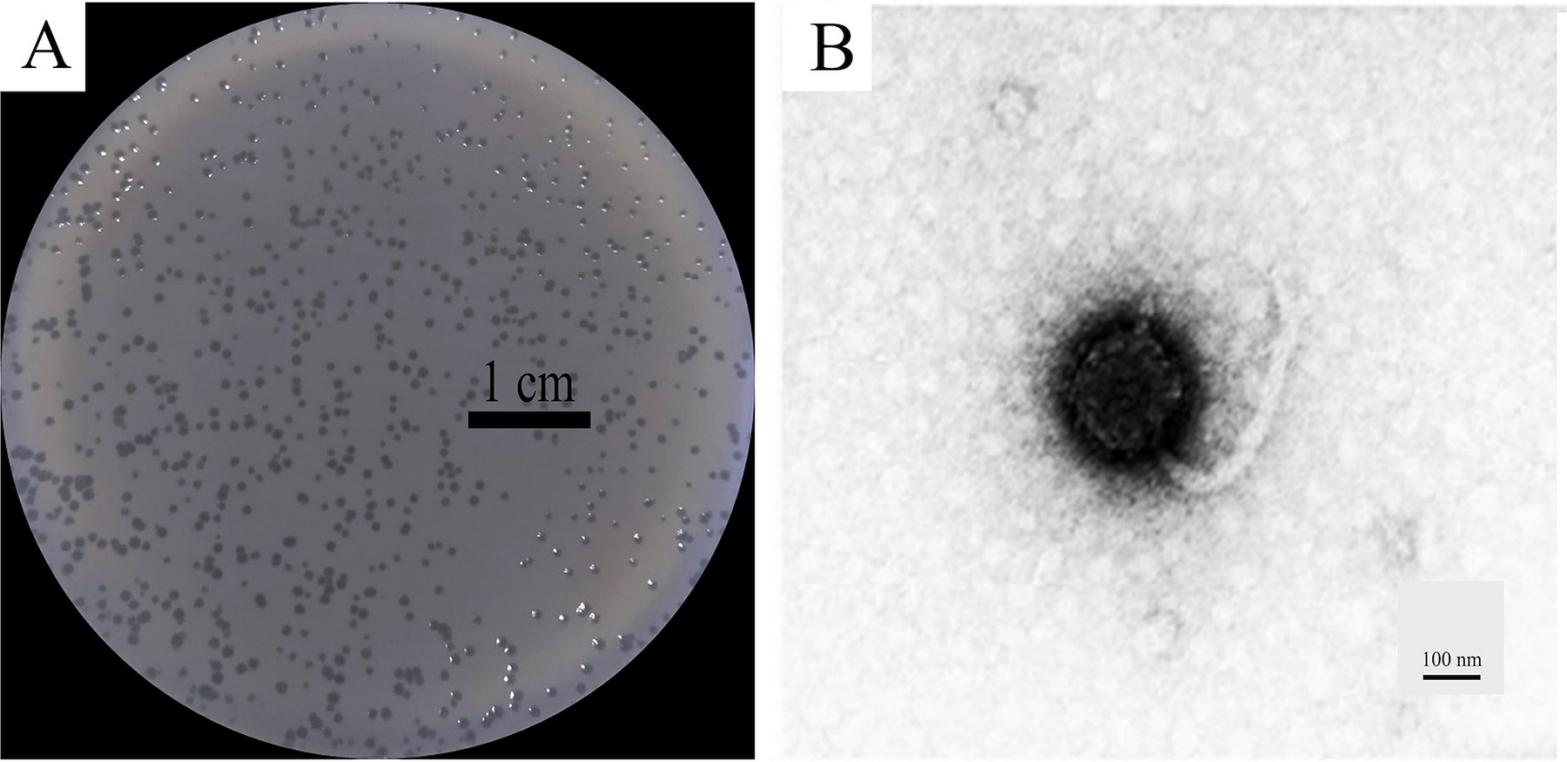
(A and B) Plaque morphology (A) and transmission electron microscopy images (B) of phage OPA17.

In this study, we isolated phage OPA17 from Anadara granosa (blood cockles) and propagated them using *V. campbellii* HY01 as a host strain. The phages showed small clear plaques with diameters of approximately 0.1 to 0.2 mm (Fig. 1A). Transmission electron microscopy revealed that OPA17 had an icosahedral head with a diameter of approximately 160 nm. A long flexible tail, approximately 320 nm long, was observed (Fig. 1B). Based on electron microscopic images with the guidelines of the International Committee on Taxonomy of Viruses (ICTV), phage OPA17 was determined to belong to the *Siphoviridae* family.

### Host specificity.

A total of 136 *Vibrio* strains in various species were used for the determination of phage host range. Among these, 35 isolates of *V. campbellii* were obtained from a previous study, and 83 isolates were isolated from shrimp larvae, water samples in hatcheries, and seawater in this study. Phage OPA17 infected 83.89% (99/118) of the *V. campbellii* isolates. Interestingly, it showed the ability to inhibited 2 clinical strains of V. parahaemolyticus and some isolates of V. vulnificus (Table 1).

**TABLE 1:** Host range of the isolated phage OPA17 against 136 strains of *Vibrio* spp.

Species (*n*)	Isolates(s)	Phage sensitivity^*a*^	Reference or source
*V. campbellii* (118)	HY01, PSU3280, PSU3282, PSU3284, PSU3285, PSU3287, PSU3288, PSU3290, PSU3294, PSU3295, PSU3296, PSU3301, PSU3306, PSU3312, PSU3313, PSU3314, PSU3316	+	27
	PSU3281, PSU3283, PSU3289, PSU3291, PSU3292, PSU3293, PSU3297, PSU3298, PSU3299, PSU3300, PSU3302, PSU3303, PSU3304, PSU3307, PSU3309, PSU3311, PSU3318, PSU3319	−	27
	PSU6075, PSU6076, PSU6077, PSU6078, PSU6079, PSU6081, PSU6082, PSU6083, PSU6084, PSU6085, PSU6086, PSU6087, PSU6088, PSU6089, PSU6090, PSU6091, PSU6092, PSU6093, PSU6094, PSU6095, PSU6096, PSU6097, PSU6098, PSU6099, PSU6100, PSU6101, PSU6102, PSU6103, PSU6104, PSU6105, PSU6106, PSU6107, PSU6108, PSU6109, PSU6110, PSU6111, PSU6112, PSU6113, PSU6114, PSU6115, PSU6116, PSU6117, PSU6118, PSU6119, PSU6120, PSU6121, PSU6122, PSU6123, PSU6124, PSU6125, PSU6126, PSU6127, PSU6128, PSU6129, PSU6130, PSU6131, PSU6132, PSU6133, PSU6134, PSU6135, PSU6136, PSU6137, PSU6138, PSU6139, PSU6140, PSU6141, PSU6142, PSU6143, PSU6144, PSU6145, PSU6146, PSU6147, PSU6148, PSU6149, PSU6150, PSU6151, PSU6152, PSU6153, PSU6154, PSU6155, PSU6156, PSU6157	+	This study
	PSU6080	−	This study
V. alginolyticus (4)	PSU6, PSU765, PSU3425, PSU3603	−	Laboratory collection
V. furnissii (1)	PSU5036	−	Laboratory collection
V. harveyi (1)	PSU15	−	Laboratory collection
V. parahaemolyticus (7)	PSU3866, PSU4413 PSU4918, PSU5124 PSU5580, PSU5591 ATCC17802	+	59
		−	57
		−	58
		−	ATCC
V. vulnificus (5)	C35, PSU039 PSU025, D22 DMST31752	+	60
		−	60
		−	Department of Medical Sciences Thailand

a +, Plaques were observed; –, no plaques were observed.

### *In vitro V. campbellii* reduction assay.

**FIG 2:**
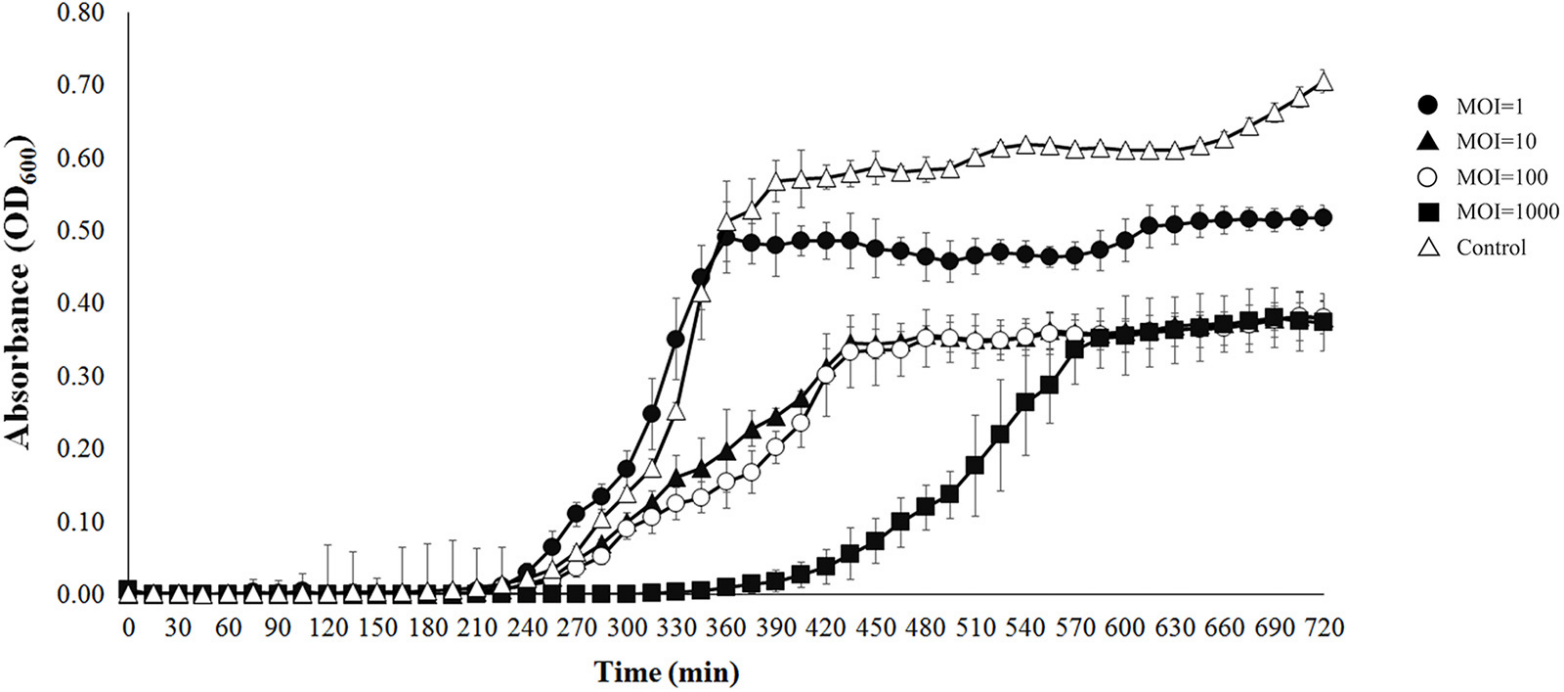
Growth curves of Vibrio campbellii HY01 in the presence of OPA17. Different MOIs (1, 10, 100, 1,000) were added to bacterial culture. The values are means ± standard deviation of three replicates.

The *in vitro* killing effect of OPA17 on *V. campbellii* HY01 was observed at different multiplicities of infection (MOIs; 1, 10, 100, and 1,000). Lysis of bacterial culture at all MOIs tested was observed through the continuous measurement of the optical density at 600 nm (OD_600_) for 720 h. In samples treated with phage OPA17, there was a decrease in optical density of *V. campbellii* HY01, and the difference in bacterial growth between the control and the treated group was observed after 4 h. The delay in bacterial growth during the exponential phase was observed in treated cultures, and the reduction of *V. campbellii* HY01 was proportional to the MOI. However, the complete inhibition was not observed using the concentration of phage tested in this study (Fig. 2).

### One-step growth curve and phage survival in ASW.

**FIG 3:**
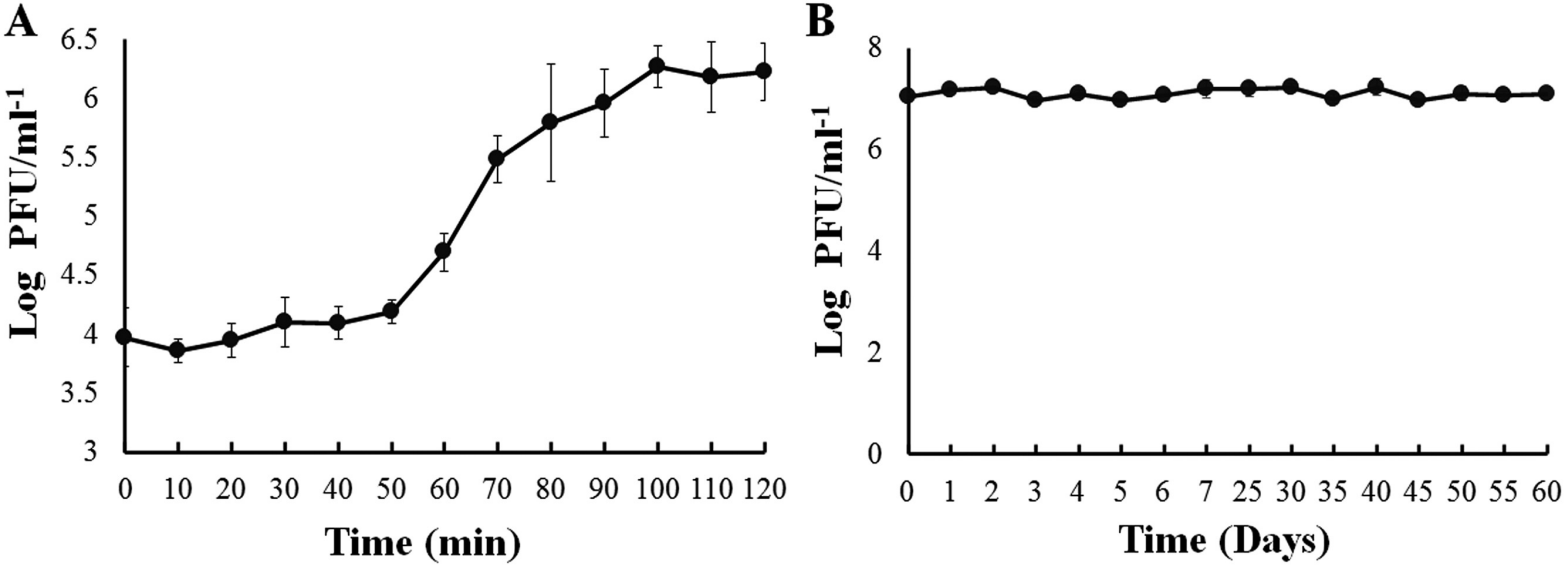
(A and B) One-step growth curve of phage OPA17 (A) and survival of phage OPA17 in artificial seawater (B). The values are means ± standard deviation of three replicates.

The one-step growth curve shows that the latent period of OPA17 was approximately 50 min with a burst size of 123 PFU/infected cell (Fig. 3A). The viability of phage OPA17 in artificial seawater (ASW) remained stable during the 60 days of the experiment (Fig. 3B).

### Eradication of preformed biofilms.

**FIG 4:**
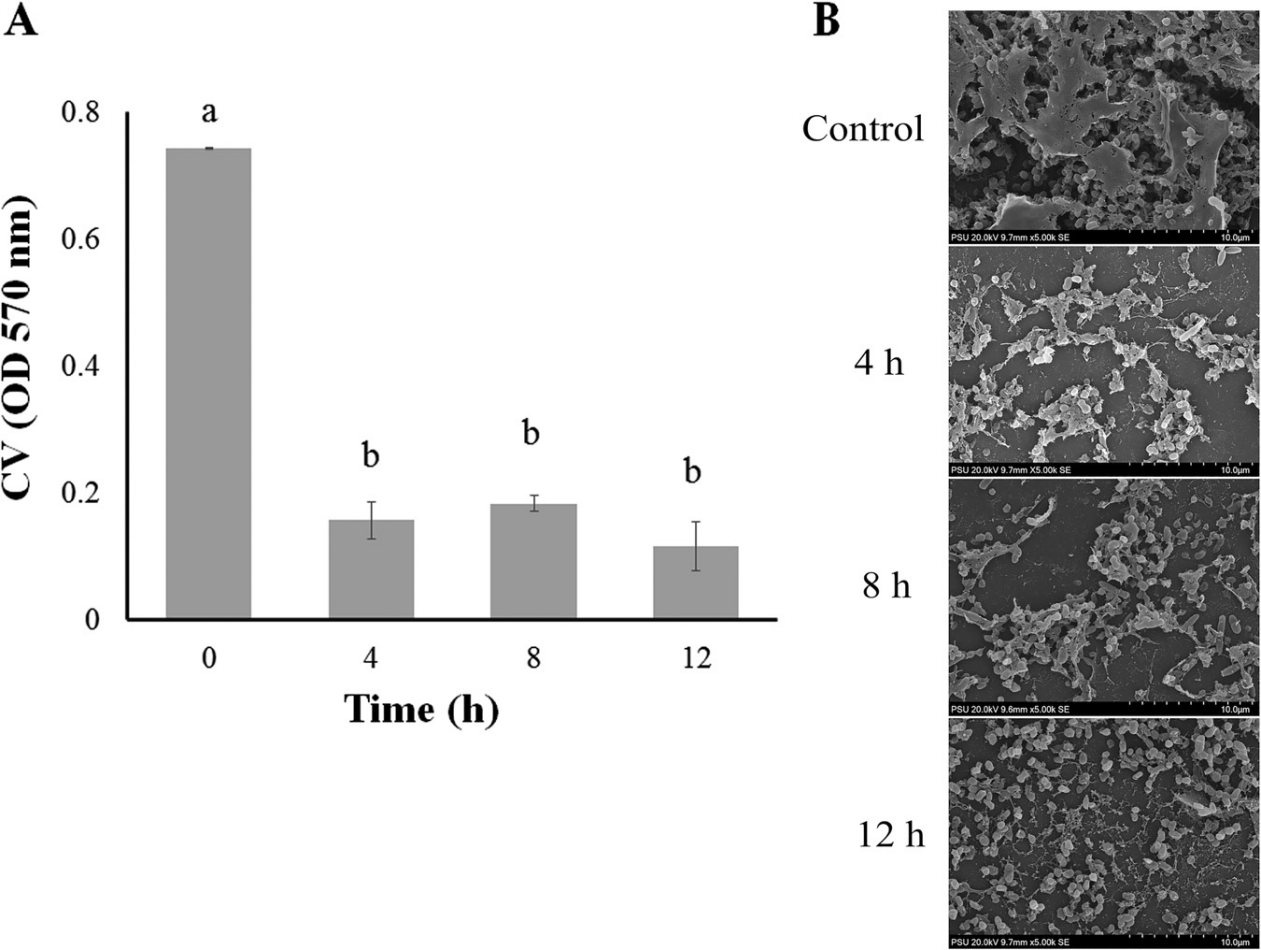
(A and B) Crystal violet staining of *V. campbellii* biofilms (A) and scanning electron microscope (SEM) images (B) of biofilm formation on glass coverslips. Samples were treated with OPA17 at an MOI of 1,000 for 4, 8, and 12 h. Different letters above the bars indicate significant differences (*P < *0.05).

*V. campbellii* biofilms were treated with OPA17 at an MOI of 1,000 and sampled at 0, 4, 8, and 12 h. The results indicated that after 4 h of exposure to OPA17, the *V. campbellii* numbers and biofilms were clearly reduced compared to the untreated controls (without phage) (Fig. 4). In addition, reductions in bacterial aggregation and clumping were observed in the treated group, especially at 12 h after phage treatment (Fig. 4B).

### Phage genome analysis.

**FIG 5:**
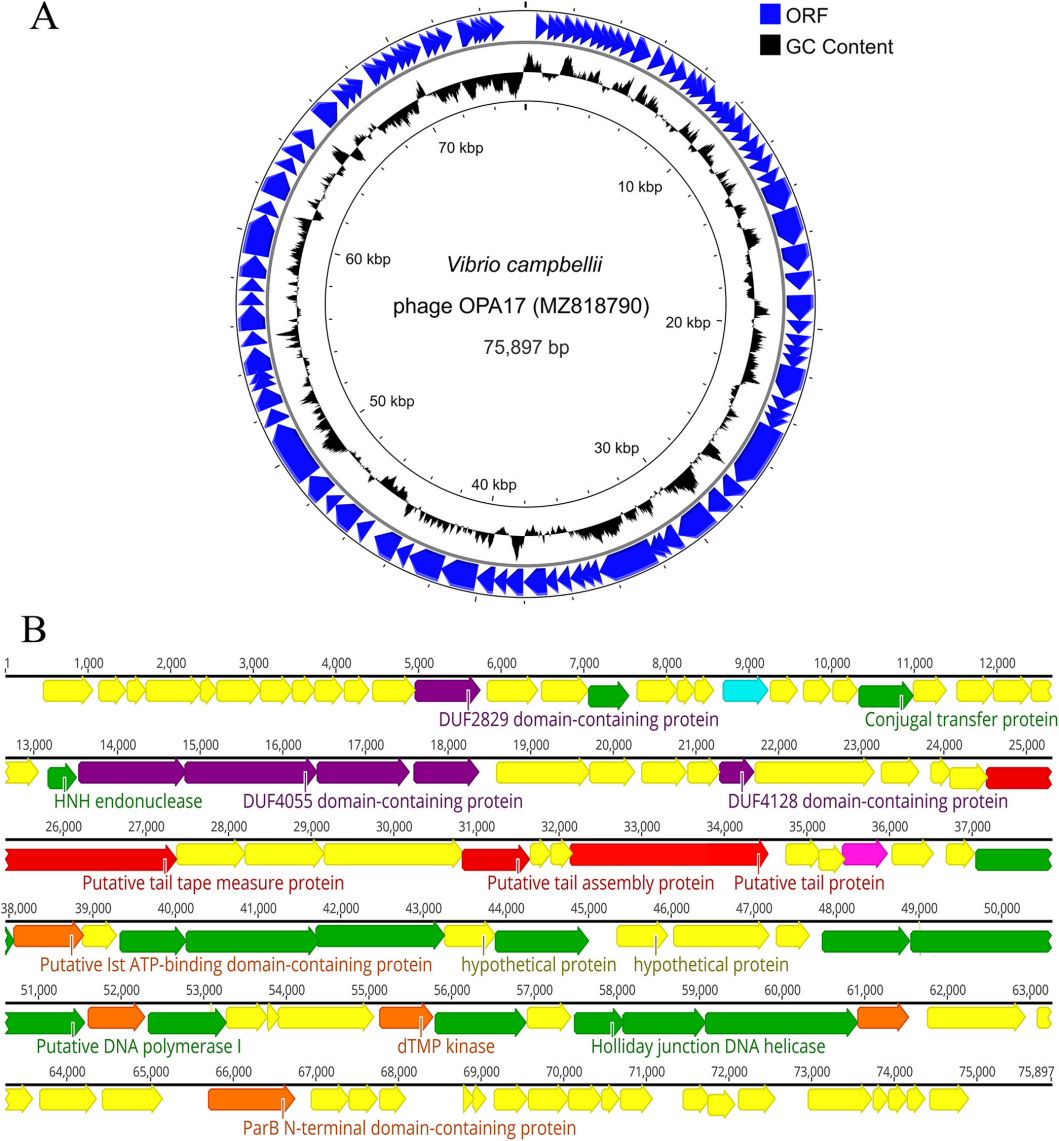
(A and B) Circular genome map (A) and genome organization (B) of phage OPA17. The predicted functional proteins are represented by different colors based on defined categories (purple, phage structural and packaging protein; red, tail assembly; green, DNA replication and modification; pink, host lysis proteins; blue, transcriptional regulator; orange, additional function; yellow, hypothetical proteins).

**FIG 6:**
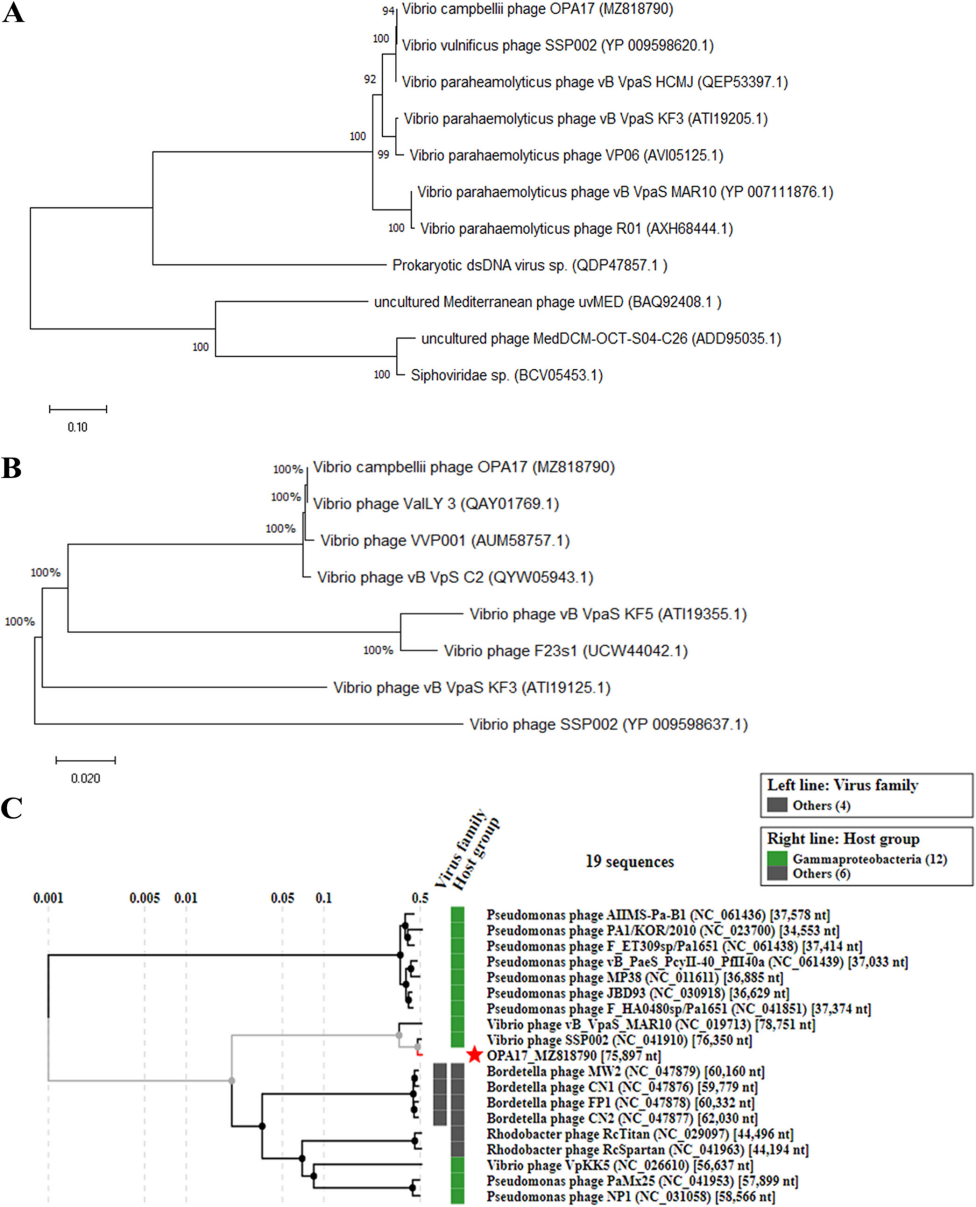
Phylogenetic relationships between OPA17 and other phages. (A and B) Amino acid sequences of the terminase large subunit (A) and putative tail protein (B) were aligned using ClustalW in MEGA X. (C) The viral proteomic tree was constructed with ViPTree.

**FIG 7:**
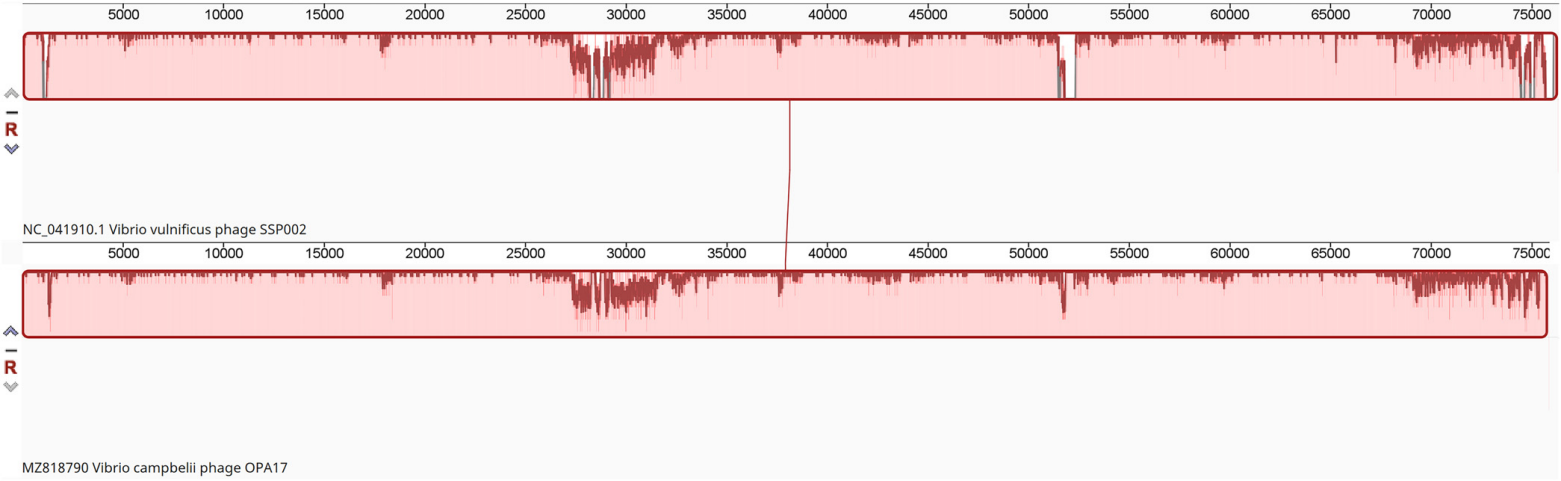
Genomic comparisons between Vibrio campbellii phage OPA17 and V. vulnificus phage SSP002 using progressiveMauve.

The genome size of OPA17 was 75,897 bp with a G+C content of 48.41% (Fig. 5A). It contains 102 predicted open reading frames (ORFs), in which 31 functional proteins could be assigned (see Table S1 in the supplemental material). These included the proteins involved in phage structure and packaging, tail assembly, DNA replication and modification, host lysis, transcriptional regulation, and additional functions (Fig. 5B). As expected, the tail sheath protein representation of a *Myoviridae* phage was not detected. The large terminal subunit, but not the putative tail assembly protein, and phage proteomes showed high similarity with *Siphoviridae* phage SSP002 of V. vulnificus (Fig. 6). The progressive MAUVE alignment showed large common homologous regions between phage OPA17 and phage SSP002. However, some gaps were identified (Fig. 7). Bioinformatics analysis of the OPA17 genome could not find any virulence/toxin genes or antibiotic resistance genes. The predicted temperate lifestyle genes, including integrase, Cro/CI repressor protein, immunity repressor, DNA partitioning protein A (ParA), and antirepressor proteins were absent in the genomes of phage OPA17. However, an ORF containing a ParB N-terminal domain was predicted. Using BACPhlip, OPA17 was confirmed as a lytic phage with 95% confidence.

### Phage therapy in *A. franciscana* nauplii infected with *V. campbellii*.

**FIG 8:**
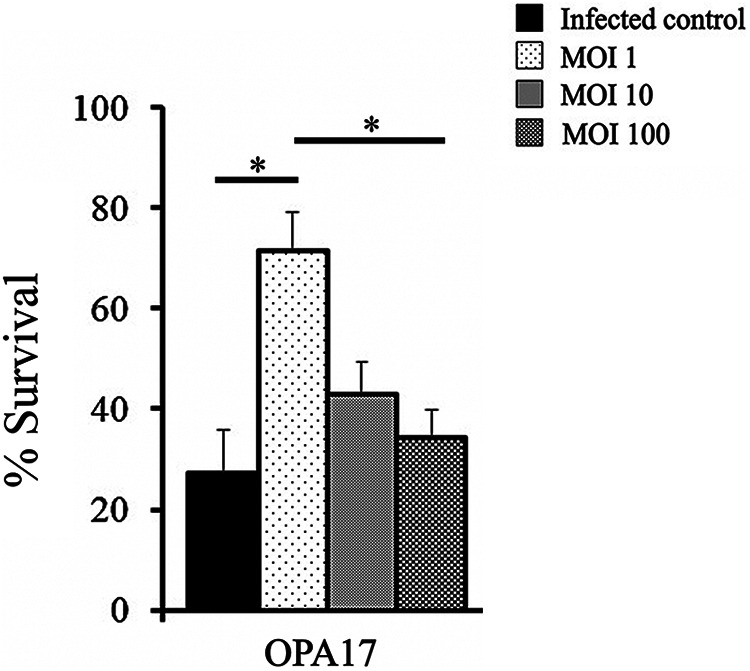
Survival of *Artemia franciscana* nauplii at 48 h postinfection with Vibrio campbellii HY01 and treatment with phage OPA17 at different MOIs. *, *P < *0.05.

The *in vivo* efficacy of phage OPA17 was examined by direct observation of *A. franciscana* nauplius survival during *V. campbellii* infection. The median lethal dose (LD_50_) of *V. campbellii* HY01 against nauplii was calculated to be 7.23 × 10^6^ CFU/mL, and this concentration was used in the challenge experiment. After 48 h of infection, the survival rate of uninfected controls and *V. campbellii*-infected controls without phage treatment was 77.87% and 27.01%, respectively. Significant (*P < *0.05) survival of phage-treated groups was observed compared with an *V. campbellii*-infected control group. The most significant increases in survival rates of phage-treated *A. franciscana* nauplii were found in nauplii treated with OPA17 at an MOI of 1 (71.43%) (Fig. 8).

## DISCUSSION

Luminous vibriosis is common bacterial disease found in shrimp hatcheries, and *V. campbellii* was recently reported as one of the pathogens associated with the disease (3–5). This study found that all luminous bacteria, except one, isolated from marine sources were confirmed as *V. campbellii*. With the advancement of molecular identification, the luminous *Vibrio* isolates from our previous study (27) were also confirmed as *V. campbellii* using hemolysin-based PCR developed by Haldar et al. (10). This correlated with a previous study which reported that *V. campbellii* was a predominant luminous pathogen found in Indian shrimp hatcheries (5). They also reported about the misidentification of *V. campbellii* as V. harveyi in their earlier study. This is not surprising, because a reference strain, *V. campbellii* BAA-1116, and a highly pathogenic *V. campbellii* strain, HY01, which was previously identified as V. harveyi, were also reclassified based on the microarray-based comparative genomic hybridization (CGH) and multilocus sequence analyses (MLSA) (6). These data indicated that *V. campbellii* is the major luminous bacterium distributed in shrimp hatcheries and marine environments in our study areas. Thus, this study addressed the selection and characterization of a *V. campbellii* lytic phage with a potential to control luminous vibriosis in shrimp hatcheries.

In this study, bacteriophage OPA17 was isolated and characterized for its efficacy against *V. campbellii* and other *Vibrios* commonly found in aquaculture environments. Based on the ICTV criteria and genomic features, phage OPA17 was classified to fall in the *Siphoviridae* family, order *Caudovirales*. According to earlier studies, OPA17 is different from other *V. campbellii* phages. In a previous study, lytic phages vB_Vc_SrVc2 and vB_Vc_SrVc9, which infect *V. campbellii*, were classified as members of the *Autographiviridae* family (4, 25). However, the potential of *Siphoviridae* phage to control of luminous vibriosis caused by V. harveyi in shrimp aquaculture has been reported in many previous studies (3, 28–30).

The ideal candidates for phage therapy should consist of phage that are able to lyse the majority of target bacteria (31). OPA17 showed high efficiency in inhibiting almost all *V. campbellii* isolates tested in this study and also infected some strains of V. parahaemolyticus and V. vulnificus. The ability of phages to infect different species within the genus *Vibrio*, especially among Harveyi clade species, has been reported in previous studies. *V. campbellii* phages vB_Vc_SrVc2 and vB_Vc_SrVc9 isolated from the hepatopancreas of shrimp with AHPND in Mexico also lysed V. parahaemolyticus and V. alginolyticus (4, 25). Other studies showed the cross-infectivity of isolated phages among Harveyi clade hosts. However, no *V. campbellii* was included in the studies. For examples, phage pVa-21 of V. alginolyticus isolated from a seawater sample in South Korea lysed one of the V. harveyi isolates (16). V. alginolyticus phages φSt2 and φGrn1 isolated from water samples in Greece infected host strains and both V. harveyi and V. parahaemolyticus (24). The cross-infectivity between V. harveyi and V. alginolyticus was also reported in Φ-6 and Φ-7 isolated from an oyster larval culture tank in Australia (23). A broad host range of *Myoviridae* vibriophage KVP40, isolated from seawater, inhibited V. parahaemolyticus, V. alginolyticus, *V. natriegens*, V. cholerae, V. mimicus, V. anguillarum, V. splendidus, V. fluvialis, and Photobacterium leiognathi. The outer membrane OmpK was suggested as the receptor for KVP40 (32). However, the flagellum was suggested as the host receptor for the narrow-host-range phage SSP002 against V. vulnificus (33, 34). The ability of phage OPA17 to inhibit other marine-pathogenic *Vibrio* species should be an interesting feature for aquaculture applications.

To assess the therapeutic potential of OPA17, the lytic ability of the phage was tested against a shrimp-pathogenic *V. campbellii* strain, HY01. In this study, real-time growth monitoring showed bacterial reduction in all phage-treated samples. However, a total elimination of *V. campbellii* was not observed. Reducing rather than eliminating the bacteria may also be advantageous because the shrimp’s immune system will be exposed to low levels of bacteria, giving them the opportunity to develop an immunity. Phage therapy is primarily limited by the emergence of resistant strains. It was reported in a previous study that, even under the same MOI, the growth stages (logarithmic or stationary) and the initial bacterial concentration (10^4^ to 10^7^) could influence the *in vitro* growth kinetics of Pseudomonas aeruginosa (35). Another study reported the effect of bacterial concentration on the emergence of the P. aeruginosa PAO1 resistance strain. In liquid culture, bacterial concentrations greater than 10^4^ CFU/mL led to phage resistance, and the resistance did not emerge in the presence of competitors (36). In a shrimp hatchery, the concentration of total and luminous vibrios is routinely monitored and should not exceed 10^3^ CFU/mL. Thus, the existence of natural *Vibrio* competitors and the acceptable *Vibrio* concentration could prevent the development of resistance in targeted isolates during phage treatment in a natural situation.

The suitable candidates for phage-based biocontrol should display a short latent period but large burst size, which were shown to be affected by the medium used and host strains. (37–41). Phage OPA17 presented a good lytic ability with a burst size and latent period of 123 PFU/cell and 50 min, respectively. The burst size and latent period of phage OPA17 were comparable to those of the earlier isolated phages. *V. campbellii* phage vB_Vc_SrVc9 was reported in the previous study to have a burst size of 78 PFU/cell and latent period of 20 min (4). In addition, phages VP-1, VP-2, and VP-3, with burst sizes of 9, 15, and 42 PFU/cell, respectively, and latent periods of 120, 90, and 40 min, respectively, were suggested for phage therapy against *Vibrio* in aquaculture (37). Additionally, phage therapy in aquaculture requires the free phage to remain viable for a period of time in the marine environment. According to the results of this study, OPA17 can survive in artificial seawater for 2 months. It is possible that the artificial seawater used in this study provides some factors (such as high salinity, neutral pH, essential elements) that help to maintain phage stability.

The ability of phage to combat biofilm-forming bacteria can be considered an advantage. Bacteriophages carry peptidoglycan hydrolases, which are effective against biofilms (42). A high phage concentration was used in biofilm studies because a low phage dose might result in insufficient biofilm removal, as bacteria in the biofilm matrix may not be adsorbed by phages. Thus, high densities of phages are suggested for biofilm disruptions. In a previous study, Yang et al. (43) indicated that phage vB_VpaP_FE11 at 10^10^ PFU/mL could significantly destroy the preformed biofilms of V. parahaemolyticus (44). Treatment of *V. campbellii* biofilm was demonstrated by phage P4F with a more than 2-log reduction of cells after treatment for 18 and 24 h (45). In this study, OPA17 at an MOI of 1,000 effectively destroyed cells and biofilm matrix of *V. campbellii* HY01. This was also observed in a previous report and was suggested to be due to the bacterial metabolism and phage proliferation within biofilm (46).

Whole-genome sequence analysis is necessary prior to selection of the candidate for safe phage therapy. It is necessary to ensure that phages do not have virulence, antibiotic resistance, or the genes encoding a lysogenic life cycle (47). In our previous study, tRNA, prophage repressor and antirepressor, and partitioning protein (ParB) sequences were found in the genome of temperate *V. campbellii* phage HY01 (48). These genes, except that encoding the ParB N-terminal domain-containing protein, were absent from the predicted ORFs of OPA17. ParB is a DNA binding protein involved in DNA separation and partitioning and has been identified not only in bacteriophages but also in bacterial chromosomes and plasmids (49). This study could not detect the sequences with homology to *parA* in OPA17 which encoded the membrane-associated ATPase essential for ParB movement. Thus, this phage behaves like lytic phages. An ORF containing ParB was reported in the genome of lytic phages SSP002 and VHP6b of V. vulnificus and V. harveyi, respectively (18, 34). The comparative genome analysis showed that OPA17 was closely related (98.90% similarity) to lytic phage SSP002 of V. vulnificus, a *Siphoviridae* phage with biocontrol activity (32–34). Phage OPA17, isolated in this study, had a broad host range, which differs from SSP002, which exhibited a narrow host range. The differences may be due to phage-host receptors. A previous study showed that the flagellum was associated with the V. vulnificus host receptors against SSP002 (33). In tailed phages, tail fibers, tail spikes, and the central tail spike are used to determined bacterial cell surface receptors (50), including lipopolysaccharide (LPS), flagella, type 4 pili, outer membrane porins, and transmembrane protein (51). The role of tail fiber assembly proteins in specific binding of the bacterial cell surface was also demonstrated in Escherichia coli phage Mu (TfaMu), which binds to LPS of its host (52). This study found that the gene encoding tail assembly protein of OPA17 (ORF 46) was highly matched to the tail assembly protein of *Vibrio* phage ValLY_3, which has broad host ranges against 5 species of *Vibrio*, V. owensii, V. parahaemolyticus, *V. natriegens*, V. metschnikovii, and V. alginolyticus (53). However, no *V. campbellii* or V. vulnificus isolates were included in that study. Further study is needed to identify the receptor that mediates the infection of OPA17.

Furthermore, the potential of OPA17 as the suitable candidate for phage therapy was performed in an *A. franciscana* nauplii model. The results of the challenge test showed that phage OPA17 at an MOI of 1 significantly increased, up to 70%, the survivability of nauplii. The efficiency of phage therapy may be due to various variabilities such as MOI, pH, salinity, NaCl, and organic matter concentration (54). Thus, these may lead to the differences observed between the *in vitro* experiment and *in vivo* treatment. In addition, the *in vivo* assay in this study showed that low concentrations of phage have a better effect than high phage concentrations, similar to the study of Quiroz-Guzmán et al., in which V. harveyi-infected brine shrimp nauplii treated with a lower dose of F12 phage (MOI, 0.1) resulted in more survival than higher phage doses (MOI, 10 and 100) (55). The high phage concentrations may cause adverse effects on the host, as Gram-negative bacteria release endotoxins when they are lysed by phages. This remains to be discussed and investigated further. The next step would be to test the effectiveness of OPA17 and OPA17 combined with another selected phage as a cocktail to control *V. campbellii* infections in shrimp hatcheries. The results of this study suggest that phage OPA17 exhibits potential as a biocontrol agent against *V. campbellii*, thereby benefiting shrimp aquaculture.

## MATERIALS AND METHODS

### Bacterial strains and growth conditions.

All bacterial strains used in this study are listed in Table 1. Three *V. campbellii* strains (HY01, PSU3282, and PSU3292) obtained from previous study (27) were used as mixed hosts for bacteriophage isolation. A pathogenic strain, *V. campbellii* HY01, obtained from shrimp that died from luminous vibriosis was used for the propagation of bacteriophage OPA17. All bacteria were maintained in 25% glycerol stock at –80°C at the Division of Biological Science, Faculty of Science, Prince of Songkla University. The isolates were grown on tryptic soy agar (TSA) (Difco, USA) supplemented with 1% NaCl at 30°C, with the exception of V. vulnificus, which was grown at 37°C.

### Isolation of *V. campbellii*.

Thiosulphate-citrate-bile salts-sucrose (TCBS) agar (Difco, USA) was used for the isolation of *V. campbellii* from shrimp (Litopenaeus vannamei) postlarval state, water samples from hatcheries, and seawater in various regions of southern Thailand. All samples were plated onto TCBS agar without an enrichment step, except for seawater samples, which were enriched with alkaline peptone water (APW). Briefly, 227.5 mL of seawater sample was transferred to 22.5 mL of 10× APW (pH 8.6) and incubated at 30°C for 6 h. Then, 0.1 mL of enrichment sample was spread onto TCBS agar. After incubation for 24 h at 30°C, the luminous colonies were picked and cultured for molecular identification. The isolates were confirmed as *V. campbellii* by PCR based on the hemolysin (*hly*) gene using primer sets and a PCR protocol published previously (10) and were used in the phage host range experiment.

### Isolation and propagation of bacteriophage.

Phage OPA17 was isolated from a blood cockle sample collected from a local market in Hat Yai, Songkhla, Thailand. The isolation method was adapted from a previous study described by Yingkajorn et al. (56). Briefly, homogenized blood cockle was transferred to 20 mL double-strength tryptic soy broth (TSB) supplemented with 1% NaCl. Then, 200 μL of early exponential phase mixed hosts was added and incubated under 100 rpm shaking at 30°C for 6 h. The mixture was centrifuged at 10,000 × *g* for 10 min, and supernatant was filtered through a 0.22-μm-pore-size syringe filter (Corning Inc., Germany). Next, 100 μL of phage filtrate was plated onto double-layered plates (24), incubated at 30°C overnight, and observed for the presence of plaque or clear zones. For phage purification, a single plaque was selected, resuspended in SM buffer (50 mM Tris-HCl, pH 7.8, 2 g of 8 mM MgSO_4_, and 5.8 g of NaCl), and the above-described steps were repeated 3 times. For phage propagation, 100 μL of purified phage was mixed with 200 μL of *V. campbellii* HY01 host in 3 mL of 0.7% soft agar. The mixture was overlaid on the TSA supplemented with 1% NaCl and incubated overnight at 30°C. Plates with lysis plaque were flooded with 3 mL of SM buffer and placed on an incubator shaker (100 rpm) at 30°C, for 6 h. The suspension was removed, centrifuged at 10,733 × *g* for 10 min, filtered through a 0.22-μm-pore-size syringe filter, and stored at 4°C until use.

### Host range determination.

The host range of phage OPA17 was tested against *V. campbellii* (*n = *118) isolates obtained in this study and from a previous report (27). Other species of vibrios used in host range determination include V. alginolyticus (*n = *4), V. parahaemolyticus (*n = *7) (57–59), V. vulnificus (*n = *5) (60), and one each of V. furnissii and V. harveyi (Table 1). The spot assay was performed to assess the lytic spectrum of the phage based on the bacterial susceptibility pattern. Briefly, 10 μL (10^9^ PFU/mL) of phage suspension was spotted on double-layered agar plate containing bacterial host (10^8^ CFU/mL). The presence of plaques or clear zones was monitored after overnight incubation at 30°C.

### Morphological characterization by transmission electron microscopy analysis.

A transmission electron microscope (TEM) was used to determine the morphological characteristics of phage OPA17. Briefly, 10 μL of purified phage lysate (10^8^ PFU/mL) was loaded onto Formvar carbon-coated grids and negatively stained with 2% phosphotungstic acid for 5 min (61). Then, the grid was examined using a TEM (JEM-100CX II; JOEL, Tokyo, Japan) operated at 80 kV with magnification of ×80,000.

### *In vitro* killing efficacy in planktonic culture.

*V. campbellii* HY01 was used as a host to determine the killing effect of OPA17 in broth culture. In brief, phage suspension at multiplicities of infection (MOIs) of 1, 10, 100, and 1,000 were added to 180 μL *V. campbellii* HY01 cultures in the early exponential growth phase (10^6^ CFU/mL). The control was inoculated without phage. A total of 200-μL mixtures were incubated in sterile 96-well plates with shaking for 12 h at 30°C, and bacterial growth (OD_600_) was monitored automatically at 15-min intervals in a microplate reader (LUMIstar Omega, Germany).

### One-step growth curve.

One-step growth curve analysis was performed as described previously (37) with some modifications. Briefly, phage was added to 10 mL of early-exponential-phase *V. campbellii* HY01 culture (10^8^ CFU/mL; MOI of 0.001) and incubated at 30°C for 5 min to allow phage adsorption. Then, the mixture was centrifuged at 10,733 × *g* for 5 min, and the supernatant containing unabsorbed free phage was discarded. Thereafter, the pellets were resuspended in 10 mL of fresh TSB plus 1% NaCl, and samples were collected at 10-min intervals during 120 min of incubation. Serial dilutions of samples were spotted on TSA plus 1**%** NaCl containing lawn of *V. campbellii* HY01 to measure the phage concentration. PFU were counted following overnight incubation at 30°C and expressed as a one-step growth curve. The experiment was repeated in triplicate. The latent period and burst size were determined as described previously (43). The burst size of phage was calculated by dividing the phage produced (PFU/mL) by the initial phage titers (PFU/mL).

### Phage survival determination.

The survival of free phage in marine water was determined according to a previous study with some modifications (37) Briefly, phage lysate (10^7^ PFU/mL) was added into 50 mL of sterile artificial seawater (ASW; 30 ppt; pH 7.1) (Mariscience International, Thailand) and incubated at 30°C for 60 days. The sample was taken at time 0 and various time points to monitor the phage survival (PFU/mL) by the double-layer agar method. The experiment was performed in triplicate.

### Biofilm eradication assay.

The effect of phage treatment on destruction of preformed biofilms was determined using crystal violet assay and scanning electron microscope (SEM) analysis. Briefly, *V. campbellii* HY01 was cultured in marine Luria-Bertani broth with glycerol medium (GmLB; 0.5% tryptone, 0.5% yeast extract, 3% sea salt, 1% glycerol, pH 6.0) (62) for 4 h at 30°C. The crystal violet assay was performed in 96-well plates. *V. campbellii* HY01 culture was inoculated into each well and incubated at 30°C for 48 h. Then, the supernatant was discarded, plate was washed with phosphate-buffered saline (PBS) to removed planktonic cells. Phage OPA17 suspension at 10^9^ PFU/mL (MOI, 1,000) was added and incubated for 4, 8, and 12 h. Then, the plate was washed with PBS and air-dried, and the biofilm was stained with 0.1% crystal violet (Merck, Virginia, USA) for 30 min. Crystal violet was dissolved in 100 μL of 95% ethanol and the OD was measured at 570 nm using a microplate reader (LUMIstar Omega, Germany).

To develop preformed biofilms for SEM analysis, 1 mL (10^6^ CFU/mL) of *V. campbellii* culture was applied to coverslips placed in a 24-well plate and incubated at 30°C for 48 h. For the biofilm eradication assay, the plate was treated with 1 mL of OPA17 phage suspension at 10^9^ PFU/mL (MOI, 1,000) for 4, 8, and 12 h. Then, coverslips were fixed in 2.5% glutaraldehyde solution for 2 h, washed with PBS and distilled water, and dehydrated through an ethanol series before being examined with an SEM (Hitachi-SU3900, Japan) (63).

### Whole-genome sequencing and bioinformatics analysis.

The phage OPA17 genome was extracted using a phage DNA isolation kit (Norgen Biotek, Ontario, Canada). The concentration and quality of the genomic DNA were evaluated using a NanoDrop spectrophotometer (Maestrogen, Inc., Nevada, USA) and agarose gel electrophoresis. The whole genome of phage OPA17 was sequenced by Macrogen, Inc., (Seoul, South Korea) using the Illumina DNA sequencer. *De novo* assembly analysis was performed at various k-mer levels with SPAdes. Open reading frames (ORFs) were predicted using various computational software in combination with GeneMark S version 4.28 (http://exon.gatech.edu/GeneMark/genemarks.cgi), Rapid Annotation using Subsystem Technology (RAST) version 2 (https://rast.nmpdr.org/), and the PHASTER (PHAge Search Tool Enhanced Release) web server. Functional annotation was scanned using the BLASTP and PSI-BLAST (https://blast.ncbi.nlm.nih.gov/Blast.cgi) algorithms against the nonredundant protein database at NCBI.

To analyze the phylogenetic relationship among phage OPA17 and other phages, phylogenetic analysis was performed using the amino acid sequences of terminase large subunit and putative tail assembly proteins. The selected sequence was aligned with ClustalW in MEGA X (64), using the neighbor-joining method with 1,000 bootstraps. In addition, the ViPTree was used to generate a virus proteomic tree based on the similarities of whole-genome sequences (65). The selected phage genomes were aligned using the progressiveMAUVE algorithm in Geneious version 9.0.4 (66). In addition, VirulenceFinder version 2.0 (67), ResFinder version 3.2 (68), and PhageLeads (69) were used to identify virulence genes, antibiotic resistance genes, and temperate phage markers, respectively. BACPHLIP (70) was used with the default parameter settings to confirm the lifestyle of phage.

### *Artemia* challenge tests.

Artemia franciscana cysts were hatched as previously described by Gomez-Gil et al. (8) and Soto-Rodriguez et al. (71) with some modifications. Briefly, 60 mg of *Artemia* cysts (CPAC Asia Products Ltd., Thailand) were incubated in 180 mL of sterile artificial seawater (ASW; 25 ppt, pH 8.6) (Mariscience International, Thailand) at 28°C with constant agitation and lighting for 16 h. The hatching quality of *Artemia* cysts was observed by evaluating the hatching percentage and hatching rate. Hatched nauplii were collected and transferred to sterile 15-mL tubes containing 10 mL of sterile ASW for further study.

The median lethal dose (LD_50_) of *V. campbellii* HY01 to *Artemia* nauplii was determined by adding *V. campbellii* HY01 into the nauplii culture water (50 nauplii/group) at a final concentration of 10^5^, 10^6^, or 10^7^ CFU/mL. The survival and LD_50_ were calculated after infection for 48 h. The experiment was performed in triplicate.

The challenge tests were performed according to the method described in reference 72 with some modifications. Briefly, 50 nauplii/group were placed in bottles with 10 mL of sterile ASW. The challenge included an uninfected control (nauplii in seawater only), positive control (infected nauplii with no phage treatment), and infected nauplii with OPA17 phage treatment. An aliquot of 200 μL of *V. campbellii* HY01 (at the LD_50_) and phages at 3 different MOIs (MOI, 1, 10, and 100) was added to the bottles. The survival was recorded at 48 h. Each treatment was performed in triplicate.

### Statistical analyses.

The data from three replicates were expressed as the mean ± standard deviation (SD). The one-way analysis of variance (ANOVA) implemented in the statistical Package for the Social Science (SPSS) version 26, at the *P < *0.05 level was used to analyze the significant differences. *Artemia* nauplii survival was analyzed using the nonparametric Kruskal-Wallis test.

### Ethics statement.

The experiment involving animals in this study was carried out in accordance with ethical guidelines and was approved by the Institutional Animal Care and Use Committee, Prince of Songkla University (2022-Sci03-007 Ref. AQ023/2022).

### Data availability.

The genome sequence of OPA17 is available under GenBank accession number MZ818790.
