# Supplementary material for: Characterization and Genome Analysis of Vibrio campbellii Lytic Bacteriophage OPA17
Source: Microbiol Spectr. 2023 Jan 31;11(2):e01623-22. doi: 10.1128/spectrum.01623-22 (PMC10101143; doi:10.1128/spectrum.01623-22)
Supplement: Supplemental file 1 — Table S1. Download spectrum.01623-22-s0001.pdf, PDF file, 0.1 MB [file spectrum.01623-22-s0001.pdf]

**Table S1** Annotation of OPA17 phage genome, ORFs, gene products, and their homology to proteins in the GenBank database investigated by BLASTP.

| ORFs | Strand | Start | Stop | Size | Protein description [organism] | Coverage (%) | *E*-value | Identity (%) | Accession no. |
|---|---|---|---|---|---|---|---|---|---|
| 1 | + | 463 | 1050 | 588 | hypothetical protein ValLY3_92 [Vibrio phage ValLY_3] | 100% | 5.00E-144 | 99.49% | QAY01813.1 |
| 2 | + | 1125 | 1463 | 339 | hypothetical protein HCMJ_2 [Vibrio phage vB_VpaS_HCMJ] | 100% | 2.00E-77 | 97.32% | QEP53372.1 |
| 3 | + | 1468 | 1692 | 225 | hypothetical protein HCMJ_3 [Vibrio phage vB_VpaS_HCMJ] | 100% | 5.00E-48 | 100.00% | QEP53468.1 |
| 4 | + | 1694 | 2362 | 669 | hypothetical protein ValLY3_89 [Vibrio phage ValLY_3] | 100% | 3.00E-166 | 100.00% | QAY01810.1 |
| 5 | + | 2352 | 2549 | 198 | Hypothetical protein FDH27_gp005 [Vibrio phage SSP002] | 100% | 7.00E-42 | 100.00% | YP_009598596.1 |
| 6 | + | 2554 | 3081 | 528 | hypothetical protein FDH27_gp006 [Vibrio phage SSP002] | 100% | 1.00E-130 | 100.00% | YP_009598597.1 |
| 7 | + | 3086 | 3466 | 381 | hypothetical protein FDH27_gp007 [Vibrio phage SSP002] | 100% | 7.00E-89 | 98.41% | YP_009598598.1 |
| 8 | + | 3468 | 3743 | 276 | hypothetical protein FDH27_gp008 [Vibrio phage SSP002] | 100% | 8.00E-63 | 100.00% | YP_009598599.1 |
| 9 | + | 3740 | 4087 | 348 | hypothetical protein ValLY3_84 [Vibrio phage ValLY_3] | 100% | 6.00E-79 | 100.00% | QAY01805.1 |

**Table S1** Annotation of OPA17 phage genome, ORFs, gene products, and their homology to proteins in the GenBank database investigated by BLASTP (continued).

| ORFs | Strand | Start | Stop | Size | Protein description [organism] | Coverage (%) | *E*-value | Identity (%) | Accession no. |
|---|---|---|---|---|---|---|---|---|---|
| 10 | + | 4097 | 4396 | 300 | hypothetical protein FDH27_gp010 [Vibrio phage SSP002] | 100% | 2.00E-69 | 100.00% | YP_009598601.1 |
| 11 | + | 4448 | 4951 | 504 | hypothetical protein KF6_008 [Vibrio phage vB_VpaS_KF6] | 100% | 5.00E-118 | 100.00% | ATI19416.1 |
| 12 | + | 4951 | 5733 | 783 | DUF2829 domain-containing protein [Vibrio phage SSP002] | 100% | 0 | 99.23% | YP_009598603.1 |
| 13 | + | 5820 | 6434 | 615 | hypothetical protein FDH27_gp013 [Vibrio phage SSP002] | 100% | 2.00E-148 | 99.51% | YP_009598604.1 |
| 14 | + | 6480 | 7052 | 573 | hypothetical protein FDH27_gp014 [Vibrio phage SSP002] | 100% | 1.00E-137 | 99.47% | YP_009598605.1 |
| 15 | + | 7052 | 7540 | 489 | putative DNA ligase [Vibrio phage vB_VpaS_KF6] | 100% | 3.00E-115 | 100.00% | ATI19420.1 |
| 16 | + | 7645 | 8127 | 483 | hypothetical protein FDH27_gp016 [Vibrio phage SSP002] | 100% | 2.00E-118 | 100.00% | YP_009598607.1 |
| 17 | + | 8124 | 8345 | 222 | hypothetical protein KF5_017 [Vibrio phage vB_VpaS_KF5] | 100% | 3.00E-48 | 100.00% | ATI19327.1 |
| 18 | + | 8345 | 8572 | 228 | hypotheticalproteinFDH27_gp018 [Vibrio phage SSP002] | 100% | 9.00E-47 | 100.00% | YP_009598609.1 |

**Table S1** Annotation of OPA17 phage genome, ORFs, gene products, and their homology to proteins in the GenBank database investigated by BLASTP (continued).

| ORFs | Strand | Start | Stop | Size | Protein description [organism] | Coverage (%) | *E*-value | Identity (%) | Accession no. |
|---|---|---|---|---|---|---|---|---|---|
| 19 | + | 8676 | 9224 | 549 | putative transcriptional regulator [Vibrio phage vB_VpaS_KF5] | 100% | 1.00E-133 | 99.45% | ATI19328.1 |
| 20 | + | 9252 | 9581 | 330 | hypothetical protein FDH27_gp020 [Vibrio phage SSP002] | 100% | 8.00E-77 | 100.00 % | YP_009598611.1 |
| 21 | + | 9649 | 9981 | 333 | hypothetical protein FDH27_gp021 [Vibrio phage SSP002] | 100% | 2.00E-76 | 100.00 % | YP_009598612.1 |
| 22 | + | 10013 | 10309 | 297 | hypothetical protein FDH27_gp022 [Vibrio phage SSP002] | 100% | 2.00E-66 | 100.00 % | YP_009598613.1 |
| 23 | + | 10320 | 10973 | 654 | conjugal transfer protein [Vibrio phage vB_VpaS_KF6] | 100% | 2.00E-164 | 100.00 % | ATI19426.1 |
| 24 | + | 10978 | 11376 | 399 | hypothetical protein VVP001_079 [Vibrio phage VVP001] | 100% | 1.00E-93 | 99.24% | AUM58779.1 |
| 25 | + | 11510 | 11956 | 447 | hypothetical protein FDH27_gp025 [Vibrio phage SSP002] | 100% | 2.00E-103 | 99.32% | YP_009598616.1 |
| 26 | + | 11958 | 12413 | 456 | hypothetical protein FDH27_gp026 [Vibrio phage SSP002] | 100% | 2.00E-109 | 100.00 % | YP_009598617.1 |

**Table S1** Annotation of OPA17 phage genome, ORFs, gene products, and their homology to proteins in the GenBank database investigated by BLASTP (continued).

| ORFs | Strand | Start | Stop | Size | Protein description [organism] | Coverage (%) | *E*-value | Identity (%) | Accession no. |
|---|---|---|---|---|---|---|---|---|---|
| 27 | + | 12406 | 13506 | 651 | hypothetical protein KF5_026 [Vibrio phage vB_VpaS_KF5] | 100% | 9.00E-159 | 100.00% | ATI19336.1 |
| 28 | + | 13166 | 13510 | 345 | HNH endonuclease [Vibrio phage SSP002] | 100% | 1.00E-80 | 100.00% | YP_009598619.1 |
| 29 | + | 13530 | 14834 | 1305 | terminase family protein [Vibrio phage SSP002] | 100% | 0 | 100.00% | YP_009598620.1 |
| 30 | + | 14821 | 16416 | 1596 | DUF4055 domain-containing protein [Vibrio phage SSP002] | 98% | 0 | 100.00% | YP_009598621.1 |
| 31 | + | 16420 | 17538 | 1119 | putative head morphogenesis domain protein [Vibrio phage SSP002] | 100% | 0 | 100.00% | YP_009598622.1 |
| 32 | + | 17584 | 18375 | 792 | putative structural protein [Vibrio phage ValLY_3] | 100% | 0 | 100.00% | QAY01783.1 |
| 33 | + | 18584 | 19711 | 1128 | hypothetical protein FDH27_gp033 [Vibrio phage SSP002] | 100% | 0 | 100.00% | YP_009598624.1 |
| 34 | + | 19725 | 20258 | 534 | hypothetical protein VVP001_069 [Vibrio phage VVP001] | 100% | 3.00E-127 | 99.44% | AUM58769.1 |
| 35 | + | 20343 | 20885 | 543 | hypothetical protein FDH27_gp035 [Vibrio phage SSP002] | 100% | 2.00E-132 | 100.00% | YP_009598626.1 |

**Table S1** Annotation of OPA17 phage genome, ORFs, gene products, and their homology to proteins in the GenBank database investigated by BLASTP (continued).

| ORFs | Strand | Start | Stop | Size | Protein description [organism] | Coverage (%) | *E*-value | Identity (%) | Accession no. |
|---|---|---|---|---|---|---|---|---|---|
| 36 | + | 20898 | 21293 | 396 | hypothetical protein KF6_031 [Vibrio phage vB_VpaS_KF6] | 100% | 3.00E-94 | 100.00% | ATI19439.1 |
| 37 | + | 21290 | 21703 | 414 | DUF4128 domain-containing protein [Vibrio phage SSP002] | 100% | 3.00E-99 | 100.00% | YP_009598628.1 |
| 38 | + | 21715 | 23157 | 1443 | hypothetical protein FDH27_gp038 [Vibrio phage SSP002] | 100% | 0 | 100.00% | YP_009598629.1 |
| 39 | + | 23248 | 23700 | 453 | hypothetical protein FDH27_gp039 [Vibrio phage SSP002] | 100% | 7.00E-106 | 100.00% | YP_009598630.1 |
| 40 | + | 23844 | 24092 | 249 | hypothetical protein FDH27_gp040 [Vibrio phage SSP002] | 100% | 1.00E-53 | 100.00% | YP_009598631.1 |
| 41 | + | 24067 | 24522 | 456 | hypothetical protein FDH27_gp041 [Vibrio phage SSP002] | 100% | 2.00E-108 | 100.00% | YP_009598632.1 |
| 42 | + | 24522 | 27368 | 2847 | putative tail tape measure protein [Vibrio phage F23s1] | 100% | 0 | 99.68% | UCW44038.1 |
| 43 | + | 27371 | 28201 | 831 | hypothetical protein ValLY3_50 [Vibrio phage ValLY_3] | 100% | 0 | 100.00% | QAY01772.1 |
| 44 | + | 28202 | 29152 | 951 | hypothetical protein ValLY3_49 [Vibrio phage ValLY_3] | 100% | 0 | 99.68% | QAY01771.1 |

**Table S1** Annotation of OPA17 phage genome, ORFs, gene products, and their homology to proteins in the GenBank database investigated by BLASTP (continued).

| ORFs | Strand | Start | Stop | Size | Protein description [organism] | Coverage (%) | *E*-value | Identity (%) | Accession no. |
|---|---|---|---|---|---|---|---|---|---|
| 45 | + | 29154 | 30833 | 1680 | hypothetical protein ValLY3_48 [Vibrio phage ValLY_3] | 100% | 0 | 98.93% | QAY01770.1 |
| 46 | + | 30833 | 31639 | 807 | putative tail assembly protein [Vibrio phage ValLY_3] | 100% | 0 | 100.00% | QAY01769.1 |
| 47 | + | 31660 | 31902 | 243 | hypothetical protein FDH27_gp047 [Vibrio phage SSP002] | 100% | 6.00E-52 | 100.00% | YP_009598638.1 |
| 48 | + | 31902 | 32147 | 246 | hypothetical protein KF5_047 [Vibrio phage vB_VpaS_KF5] | 100% | 4.00E-54 | 100.00% | ATI19357.1 |
| 49 | + | 32128 | 34683 | 2556 | putative tail protein [Vibrio phage VVP001] | 99% | 0% | 99.53% | AUM58754.1 |
| 50 | + | 34742 | 35164 | 423 | hypothetical protein FDH27_gp050 [Vibrio phage SSP002] | 100% | 1.00E-94 | 100.00% | YP_009598641.1 |
| 51 | + | 35142 | 35456 | 315 | hypothetical protein FDH27_gp051 [Vibrio phage SSP002] | 100% | 2.00E-67 | 100.00% | YP_009598642.1 |
| 52 | + | 35422 | 35970 | 549 | transglycosylase domain-containing protein [Vibrio phage vB_VpaS_KF5] | 100% | 8.00E-133 | 99.45% | ATI19361.1 |

**Table S1** Annotation of OPA17 phage genome, ORFs, gene products, and their homology to proteins in the GenBank database investigated by BLASTP (continued).

| ORFs | Strand | Start | Stop | Size | Protein description [organism] | Coverage (%) | *E*-value | Identity (%) | Accession no. |
|---|---|---|---|---|---|---|---|---|---|
| 53 | + | 36026 | 36517 | 492 | hypothetical protein FDH27_gp053 [Vibrio phage SSP002] | 100% | 7.00E-114 | 97.55% | YP_009598644.1 |
| 54 | + | 36676 | 37029 | 354 | hypothetical protein KF5_054 [Vibrio phage vB_VpaS_KF5] | 89% | 3.00E-72 | 100.00% | ATI19364.1 |
| 55 | + | 37032 | 38051 | 1020 | putative conjugative transposon protein [Vibrio phage F23s1] | 100% | 0 | 98.23% | UCW44050.1 |
| 56 | + | 38056 | 38892 | 837 | putative Ist ATP-binding domain-containing protein [Vibrio phage vB_VpaS_KF5] | 100% | 0 | 100.00% | ATI19366.1 |
| 57 | + | 38876 | 39298 | 423 | hypothetical protein FDH27_gp057 [Vibrio phage SSP002] | 100% | 9.00E-99 | 99.29% | YP_009598648.1 |
| 58 | + | 39339 | 40136 | 798 | putative DNA polymerase I [Vibrio phage SSP002] | 100% | 0 | 100.00% | YP_009598649.1 |
| 59 | + | 40136 | 41746 | 1611 | putative DNA helicase [Vibrio phage SSP002] | 100% | 0 | 99.81% | YP_009598650.1 |
| 60 | + | 41709 | 43259 | 1551 | putative helicase [Vibrio phage vB_VpaS_HCMJ] | 100% | 0 | 100.00% | QEP53429.1 |
| 61 | + | 43260 | 43877 | 618 | hypothetical protein KF5_061 [Vibrio phage vB_VpaS_KF5] | 100% | 5.00E-150 | 99.02% | ATI19371.1 |

**Table S1** Annotation of OPA17 phage genome, ORFs, gene products, and their homology to proteins in the GenBank database investigated by BLASTP (continued).

| ORFs | Strand | Start | Stop | Size | Protein description [organism] | Coverage (%) | *E*-value | Identity (%) | Accession no. |
|---|---|---|---|---|---|---|---|---|---|
| 62 | + | 43874 | 45004 | 1131 | DNA primase [Vibrio phage vB_VpaS_KF6] | 100% | 0 | 100.00% | ATI19466.1 |
| 63 | + | 45340 | 45963 | 624 | hypothetical protein KF6_059 [Vibrio phage vB_VpaS_KF6] | 100% | 1.00E-155 | 100.00% | ATI19467.1 |
| 64 | + | 46035 | 47189 | 1155 | hypothetical protein VVP001_038 [Vibrio phage VVP001] | 100% | 0 | 100.00% | AUM58738.1 |
| 65 | + | 47268 | 47669 | 402 | hypothetical protein FDH27_gp065 [Vibrio phage SSP002] | 100% | 2.00E-96 | 100.00% | YP_009598656.1 |
| 66 | + | 47832 | 48893 | 1062 | putative DNA polymerase III beta subunit [Vibrio phage SSP002] | 100% | 0 | 100.00% | YP_009598657.1 |
| 67 | + | 48896 | 51550 | 2655 | putative DNA polymerase I [Vibrio phage SSP002] | 100% | 0 | 100.00% | YP_009598658.1 |
| 68 | + | 51599 | 52282 | 684 | thymidylate synthase [Vibrio phage vB_VpaS_KF6] | 100% | 2.00E-171 | 99.56% | ATI19472.1 |
| 69 | + | 52334 | 53269 | 936 | putative DNA polymerase III subunit gamma and tau [Vibrio phage vB_VpaS_KF6] | 100% | 0 | 99.68% | ATI19473.1 |
| 70 | + | 53273 | 53764 | 492 | hypothetical protein FDH27_gp070 [Vibrio phage SSP002] | 100% | 1.00E-114 | 100.00% | YP_009598661.1 |

**Table S1** Annotation of OPA17 phage genome, ORFs, gene products, and their homology to proteins in the GenBank database investigated by BLASTP (continued).

| ORFs | Strand | Start | Stop | Size | Protein description [organism] | Coverage (%) | *E*-value | Identity (%) | Accession no. |
|---|---|---|---|---|---|---|---|---|---|
| 71 | + | 53766 | 53906 | 141 | hypothetical protein [Vibrio phage F23s1] | 100% | 2.00E-25 | 100.00% | UCW44066.1 |
| 72 | + | 53900 | 55048 | 1149 | hypothetical protein KF6_068 [Vibrio phage vB_VpaS_KF6] | 99% | 0 | 100.00% | ATI19476.1 |
| 73 | + | 55128 | 55763 | 636 | dTMP kinase [Vibrio phage SSP002] | 100% | 1.00E-154 | 99.53% | YP_009598664.1 |
| 74 | + | 55791 | 56894 | 1104 | DNA recombination/repair protein RecA [Vibrio phage SSP002] | 100% | 0 | 100.00% | YP_009598665.1 |
| 75 | + | 56908 | 57435 | 528 | hypothetical protein HCMJ_76 [Vibrio phage vB_VpaS_HCMJ] | 100% | 6.00E-129 | 99.43% | QEP53444.1 |
| 76 | + | 57485 | 58075 | 591 | holliday junction DNA helicase [Vibrio phage vB_VpaS_KF6] | 100% | 6.00E-142 | 100.00% | ATI19480.1 |
| 77 | + | 58072 | 59061 | 990 | putative DNA repair exonuclease [Vibrio phage VVP001] | 100% | 0 | 100.00% | AUM58725.1 |
| 78 | + | 59061 | 60908 | 1848 | putative exonuclease [Vibrio phage ValLY_3] | 100% | 0 | 100.00% | QAY01741.1 |
| 79 | + | 60910 | 61527 | 618 | putative 6-pyruvoyl tetrahydropterin synthase [Vibrio phage vB_VpaS_HCMJ] | 100% | 4.00E-150 | 100.00% | QEP53448.1 |

**Table S1** Annotation of OPA17 phage genome, ORFs, gene products, and their homology to proteins in the GenBank database investigated by BLASTP (continued).

| ORFs | Strand | Start | Stop | Size | Protein description [organism] | Coverage (%) | *E*-value | Identity (%) | Accession no. |
|---|---|---|---|---|---|---|---|---|---|
| 80 | + | 61752 | 62936 | 1185 | hypothetical protein ValLY3_14 [Vibrio phage ValLY_3] | 100% | 0 | 100.00% | QAY01739.1 |
| 81 | + | 63080 | 63574 | 495 | hypothetical protein FDH27_gp081 [Vibrio phage SSP002] | 100% | 3.00E-118 | 99.39% | YP_009598672.1 |
| 82 | + | 63667 | 64353 | 687 | hypothetical protein FDH27_gp082 [Vibrio phage SSP002] | 100% | 7.00E-168 | 99.56% | YP_009598673.1 |
| 83 | + | 64426 | 65145 | 720 | hypothetical protein FDH27_gp083 [Vibrio phage SSP002] | 100% | 5.00E-173 | 100.00% | YP_009598674.1 |
| 84 | + | 65702 | 66748 | 1047 | ParB N-terminal domain-containing protein [Vibrio phage SSP002] | 100% | 0 | 100.00% | YP_009598675.1 |
| 85 | + | 66948 | 67409 | 462 | hypothetical protein FDH27_gp085 [Vibrio phage SSP002] | 100% | 6.00E-111 | 100.00% | YP_009598676.1 |
| 86 | + | 67403 | 67741 | 339 | hypothetical protein FDH27_gp086 [Vibrio phage SSP002] | 100% | 2.00E-77 | 99.11% | YP_009598677.1 |
| 87 | + | 67771 | 68091 | 321 | hypothetical protein KF6_083 [Vibrio phage vB_VpaS_KF6] | 100% | 1.00E-72 | 100.00% | ATI19491.1 |

**Table S1** Annotation of OPA17 phage genome, ORFs, gene products, and their homology to proteins in the GenBank database investigated by BLASTP (continued).

| ORFs | Strand | Start | Stop | Size | Protein description [organism] | Coverage (%) | *E*-value | Identity (%) | Accession no. |
|---|---|---|---|---|---|---|---|---|---|
| 88 | + | 68784 | 68912 | 129 | hypothetical protein FDH27_gp088 [Vibrio phage SSP002] | 100% | 1.00E-20 | 92.86% | YP_009598679.1 |
| 89 | + | 68909 | 69061 | 153 | hypothetical protein KF3_052 [Vibrio phage vB_VpaS_KF3] | 100% | 5.00E-28 | 100.00% | ATI19168.1 |
| 90 | + | 69162 | 69554 | 393 | hypothetical protein KF3_053 [Vibrio phage vB_VpaS_KF3] | 100% | 6.00E-90 | 100.00% | ATI19169.1 |
| 91 | + | 69569 | 70057 | 489 | hypothetical protein KF6_086 [Vibrio phage vB_VpaS_KF6] | 100% | 1.00E-116 | 99.38% | ATI19494.1 |
| 92 | + | 70060 | 70464 | 405 | hypothetical protein KF6_087 [Vibrio phage vB_VpaS_KF6] | 100% | 9.00E-95 | 100.00% | ATI19495.1 |
| 93 | + | 70457 | 70681 | 225 | hypothetical protein KF6_088 [Vibrio phage vB_VpaS_KF6] | 100% | 2.00E-48 | 100.00% | ATI19496.1 |
| 94 | + | 70695 | 71072 | 378 | hypothetical protein KF6_089 [Vibrio phage vB_VpaS_KF6] | 100% | 7.00E-88 | 99.20% | ATI19497.1 |
| 95 | + | 71441 | 71719 | 279 | hypothetical protein KF6_090 [Vibrio phage vB_VpaS_KF6] | 100% | 1.00E-62 | 100.00% | ATI19498.1 |
| 96 | + | 71748 | 72074 | 327 | hypothetical protein KF6_091 [Vibrio phage vB_VpaS_KF6] | 100% | 5.00E-76 | 100.00% | ATI19499.1 |

**Table S1** Annotation of OPA17 phage genome, ORFs, gene products, and their homology to proteins in the GenBank database investigated by BLASTP (continued).

| ORFs | Strand | Start | Stop | Size | Protein description [organism] | Coverage (%) | *E*-value | Identity (%) | Accession no. |
|---|---|---|---|---|---|---|---|---|---|
| 97 | + | 72120 | 72563 | 444 | hypothetical protein KF6_092 [Vibrio phage vB_VpaS_KF6] | 100% | 2.00E-105 | 100.00% | ATI19500.1 |
| 98 | + | 72956 | 73735 | 780 | hypothetical protein [Vibrio phage vB_VpS_C2] | 100% | 0 | 99.23% | QYW05996.1 |
| 99 | + | 73751 | 73933 | 183 | hypothetical protein F862_gp105 [Vibrio phage vB_VpaS_MAR10] | 100% | 4.00E-37 | 100.00% | YP_007111951.1 |
| 100 | + | 73935 | 74156 | 222 | hypothetical protein [Vibrio phage vB_VpS_C2] | 100% | 8.00E-47 | 100.00% | QYW05998.1 |
| 101 | + | 74158 | 74373 | 216 | hypothetical protein [Vibrio phage vB_VpS_C2] | 100% | 4.00E-44 | 100.00% | QYW05999.1 |
| 102 | + | 74431 | 74907 | 477 | hypothetical protein [Vibrio phage vB_VpS_C2] | 100% | 6.00E-114 | 98.73% | QYW06000.1 |
